# Study on image data cleaning method of early esophageal cancer based on VGG_NIN neural network

**DOI:** 10.1038/s41598-022-18707-6

**Published:** 2022-08-22

**Authors:** Zhengwen Li, Runmin Wu, Tao Gan

**Affiliations:** 1 College of Life Science and Technology, University of Electronic Science and Technology of China, Chengdu, 611731 China; 2 School of Education, Sichuan Open University of China, Chengdu, 610031 China; 3 West China Hospital, Sichuan University, Chengdu, 611731 China

**Keywords:** Cancer imaging, Oesophageal cancer, Cancer screening

## Abstract

In order to clean the mislabeled images in the esophageal endoscopy image data set, we designed a new neural network VGG_NIN. Based on the new neural network structure, we developed a method to clean the mislabeled images in the esophageal endoscopy image data set. To verify the effectiveness of the proposed method, we designed two experiments using 3835 esophageal endoscopy images provided by West China Hospital of Sichuan University. The experimental results showed that the proposed method could clean about 93% of the mislabeled images in the data set, which was the first time in the cleaning of esophageal endoscopy image data set. Finally, in order to verify the generalization ability of this method, we cleaned the Kaggle open cat and dog data set, and cleaned out about 167 mislabeled images. Therefore, the proposed method can effectively screen the mislabeled images in the esophageal endoscopy image data set and has good generalization ability, which can provide great help for the development of high-performance gastrointestinal endoscopy image analysis model.

## Introduction

About 455,800 people worldwide are diagnosed with esophageal cancer each year,

and about 400,200 people die from esophageal cancer every year. The incidence of esophageal cancer is the seventh in the world, and the mortality rate is the sixth^1,2^. Studies have shown that the 5-year survival rate of advanced esophageal cancer is only 18%, while the 5-year survival rate of early esophageal cancer is more than 95%, so the early diagnosis of esophageal cancer has important clinical significance^3,4^. At present, the examination of esophageal cancer is usually performed through direct observation of gastroscopy and then biopsy to confirm the tumor stage. The accuracy of the examination results depends on the clinical experience of doctors, fatigue degree and other subjective factors. Computer-assisted diagnosis of early esophageal cancer (such as automatic classification and segmentation of esophageal diseases, etc.) provides a new approach to improve the accuracy of early diagnosis of esophageal cancer and reduce the burden of physicians^5–9^.

Recent studies have shown that deep learning has become the mainstream technology for the construction of high-performance computer-assisted early esophageal cancer diagnosis model. However, the deep learning model requires a large number of accurately labeled esophageal endoscopy image training. Large amount of annotated image data sets are difficult to obtain and are prone to annotation errors. The sources of mislabeled images can be classified into the following four categories: (1) Insufficient information, unable to provide reliable labels, such as poor image quality; (2) Incorrect labeling of experts; (3) Crowdsourcing, that is, multiple experts label differently; (4) Errors occurred during automatic labeling of neural network model^10^. Since inaccurate image labeling will have a great negative impact on the training of high-performance deep learning models^11,12^, it is necessary to develop esophageal endoscopy image data cleansing methods.

Inaccurate image data sets can be divided into two types of mislabeled images^13^: The first type is label error, that is, the real category of sample image is mislabeled, called label noise image; The other is that the image category label is correct but the image background is interfered by noise, that is, the image background is too complex and the target to be recognized is blurred or remote or too small in the image, so it is difficult to accurately mark its real type, which is not conducive to the training model^14^. It is called background noise image. Processing label noise image method has two main principles, one is to identify or modified or discarded may be misleading label sample training data^15,16^, another is to enhance the robustness of neural network model, make certain errors tag data to the model of training content does not affect or affected, such as the loss function method, data weighting method, yuan learning, etc.^15^. In the existing natural image cleaning methods, most of them only focus on the cleaning of label noise^17–23^, while others focus on the cleaning of label noise and background noise^14,24–29^. At present, most model training methods require a part of the data annotation is completely correct, that is, in the training model needs a clean data set, in the natural image data set cleaning has achieved a good effect, but the calculation is large, it is not convenient to extend to other data sets. In addition, the existing methods of cleaning label noise and background noise have not been applied to the field of esophageal endoscopy image processing. Due to the large difference in features between esophageal endoscopy images and natural images, this paper developed a cleaning method based on inaccurate supervised learning for esophageal early cancer and non-early cancer image data sets for esophageal endoscopy images, with the main contributions as follows:

1. IMPROVED the design of a new neural network VGG_NIN, so that it has the ability to quickly clean abnormal data. This method has been verified to have good results in natural image data sets^30^.
2. A new automatic cleaning method for label noise and background noise images is proposed. This method can quickly clean the image data set containing label noise and background noise, and does not require a completely clean data set.
3. The cleaning accuracy of esophageal endoscopy image data sets containing different proportions of mislabeled images was explored.
4. For the first time, abnormal data were cleaned in the inaccurate data set of esophageal endoscopy images.

## Materials and methods

### Material

#### Esophageal endoscopy image data

The dataset included 2801 endoscopic images of 672 patients with early esophageal cancer (hereinafter referred to as early esophageal cancer images), 1041 endoscopic images of 260 patients with non-early esophageal cancer (hereinafter referred to as non-early esophageal cancer images), 3711 images of esophagitis in 823 patients, and 725 images of gastric disease in 213 patients. The image data sets of early esophageal cancer and non-early esophageal cancer were used to clean the wrong images and the subsequent data sets for image classification, while the images of esophagitis and gastropathy were used to make the mislabeled images in the process of cleaning the inaccurate data sets. All images were provided by the Department of Digestive Endoscopy, West China Hospital, Sichuan University. The OlympusgIF-Q260 and Q290 gastroscopy were used for image collection. The lesions of all patients were confirmed by biopsy and examined by at least two clinicians. Cat and dog datasets involved in the paper are in the public dataset. https://www.kaggle.com/c/dogs-vs-cats/data. The patient image dataset is not publicly available and can be available from the corresponding author on reasonable request.

In accordance with the ethical requirements, the analysis of all tissue samples was performed in an anonymized manner. All esophageal endoscopy image data sets used were approved by the Medical Ethics Review Committee of West China Hospital and UESTC, and Informed consent was obtained from all patients and/or their legal guardians. Human participants' names and other HIPAA identifiers have be removed from all sections of the manuscript. All methods were performed in accordance with the relevant guidelines.

Early esophageal cancer and esophageal cancer early images, a total of 3835 pieces, the data set may contain tag errors or background noise in the image, in order to research behind early esophageal carcinoma and the early of class work, we according to the patient image data set for the classification, selection of about 70% or so of the image as the training set, about 15% of the image as a validation set, About 15% of the images were in the test set, and each patient's image was in only one set. The final training set contained 2016 images of early esophageal cancer and 731 images of non-early esophageal cancer, the validation set contained 371 images of early esophageal cancer and 155 images of non-early esophageal cancer, and the test set contained 410 images of early esophageal cancer and 152 images of non-early esophageal cancer. The specific information is shown in Table 1.

**Table 1:** Statistical table of esophageal endoscopy image data set used in this study.

	Train		Validation		Test	
	Image	Patient	Image	Patient	Image	Patient
EEC	2016	483	371	93	410	96
N-EEC	731	173	155	42	152	45
ALL	2747	656	526	135	562	141

In the process of cleaning the inaccurate data set, we only cleaned the images that might be wrong in the above training set and verification set. The images in the test set were repeatedly checked by multiple physicians to ensure that every image was correct, and no cleaning was needed.

#### Kaggle computer vision contest opens data sets

Kaggle is a platform for developers and data scientists to host machine learning contests, host databases, and write and share code. This dataset contains 12,500 images of cats and 12,500 images of dogs, and a total of 25,000 images of cats and dogs, which are used to verify the generalization ability of the algorithm in this paper to clean wrong images.

### Image cleaning method

The programming language implemented in this study is Python 3.6.4 and the deep learning library is PyTorch 1.0.0 (https://pytorch.org/). All experiments were carried out on the server based on Ubuntu 16.04.6LTS (GNU/Linux 4.8.0-36-Generic X86_64), which was equipped with Nvidia GeForce RTX2080Ti and 11G graphics processing units.

**Figure 1:**
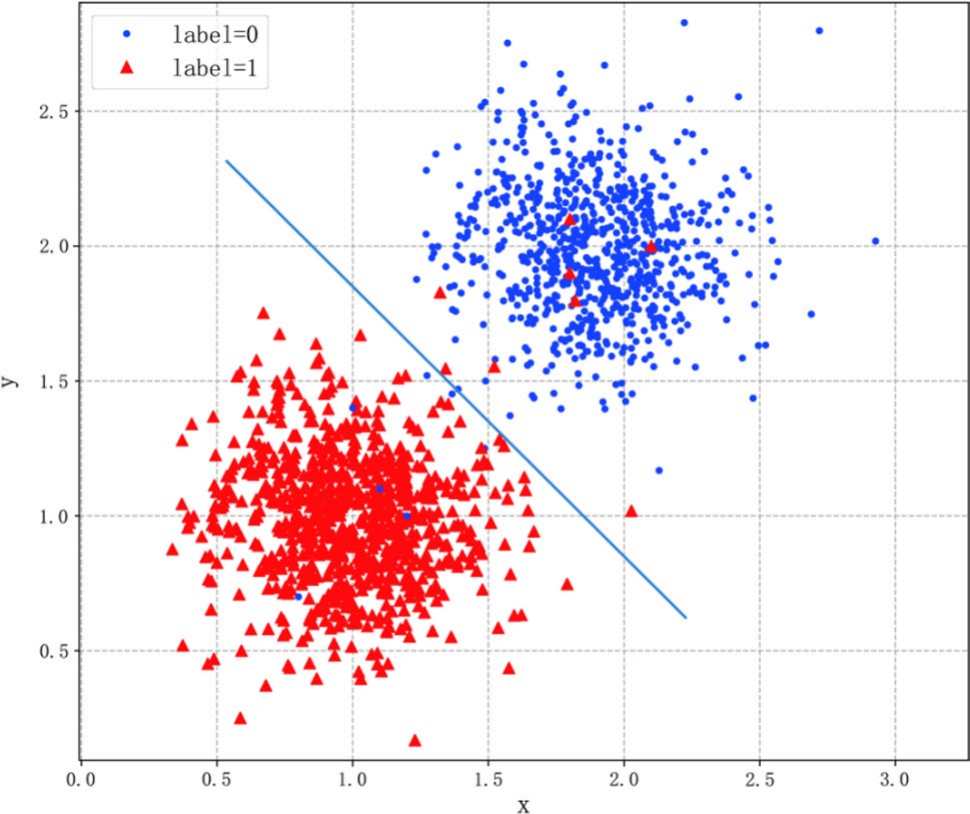
The points located in the low-density area near the dividing line are most prone to misclassification in the classification process. Most of them are low-quality images with background noise, while the wrong points located in the high-density area are mislabeled points.

The image data set cleaning in this study is based on an assumption in cluster analysis: that is, the boundary of the clustering problem must be located in the low-density region^31^. For binary image classification problem, when training the classification model, the classification model first learns the common features of each category from the high density region of the data set, and then classifies based on these common features. The main data sources causing classification errors are indistinguishable data in low-density areas and mislabeled data in high-density areas (Fig. 1), which are labeled noise data or background noise data. Therefore, if this part of data is extracted and deleted from the data set, the classification accuracy will be improved^30^.

### Image cleaning model

Neural Network VGG16_bn^32^ is a mature neural Network. Neural Network OF NIN (Network in Network)^33^ uses 1X1 convolution kernel for the first time. 1X1 convolution kernel can reduce the dimension of neural Network, improve the running speed of neural Network and improve classification accuracy. In this paper, combining the advantages of VGG16_bn neural Network and NIN (Network in Network) neural Network, we delete the full connection layer of VGG16_bn, add two 1 × 1 convolution layer and pooling layer, and finally name this neural Network as VGG_NIN neural Network. VGG_NIN neural network has 19,982,402 parameters, which is far less than that of VGG16_bn neural network with 134,277,186 parameters. Finally, an error image automatic cleaning module is added after the classification layer, which can automatically clean out the image identified by the neural network.

### Model training

In order to screen out as many wrong images as possible, the algorithm needs to repeatedly screen the data set. The specific algorithm steps are as follows:

1. All image data sets to be cleaned are used as training sets for training VGG_NIN neural network, and screening is stopped after training the specified batch.
2. The trained VGG_NIN neural network is used to classify all images in the training set, and the misclassified images are deleted from the training set.
3. Repeat steps (1) and (2).
4. When the proportion of possible error images screened is lower than the specified threshold, the screening is stopped^30^.

In the process of training the cleaning model of inaccurate data set, AdamW^34^ was selected as the optimizer of the model, and the variable learning rate and cross entropy loss function were adopted. Each batch of 32 images were trained, and the pixel size of each image was 224 × 224. The selected training model is VGG_NIN neural network. Fixed random seeds were selected in the process of training and cleaning error image model and classification model, so that the results of each experiment can be compared to a certain extent. The model training parameters are shown in Table 2.

**Table 2:** Parameters related to model training.

Image size	Loss function	Model optimizer	Image enhancement or not
224 × 224	Cross entropy loss function	AdamW	No

In the process of training the cleaning model of inaccurate data sets, AdamW^27^ is selected as the optimizer of the model and the variable learning rate and cross entropy loss function are adopted. If the loss function does not decrease every 200 batches, the learning rate is halved to improve cleaning accuracy. 32 images were trained in each batch, and the pixel size of each image was 224 × 224. The selected training model is VGG_NIN neural network. In the process of training the cleaning error image model and the classification model, we choose fixed random seeds, so that the results of each experiment can be compared to a certain extent.

Since inaccurate image data sets contain false images including label noise and background noise images, and the focus of this study is the cleaning of the early and non-early cancer data sets of esophageal endoscopy images, combined with the characteristics of esophageal endoscopy image data sets, label noise is subdivided into the following two types:

(1) Intraclass label noise images, that is, the images with mislabeled labels in the two types of images, for example, early esophageal cancer is labeled as non-early cancer, and non-early cancer is labeled as early cancer. Such label noise images are called intraclass label noise.
(2) External label noise images, that is, the images outside the binary image data set are labeled as the images of the data set. For example, the binary classification we do is premature esophageal cancer and non-premature esophageal cancer, but part of the label noise images are endoscopic gastroesophageal disease images. Such label noise images are called external label noise.

Due to esophageal endoscope image error in the data set is mainly within the class label image noise, image is early esophageal carcinoma wrong labeling for esophageal cancer early image, the early carcinoma image error in early image, so the esophagus endoscopic image data set label noise is main kinds of cleaning cleaning tags within the noise image.

### Performance evaluation indicators

In this study, the indexes for evaluating the image cleaning effect in inaccurate supervision are as follows:

1. The percentage of the number of true Error images (ATW) in the number of ALL images (ALL) in the data set is called Error rate and is expressed by (Eq. (1)). It reflects the proportion of the original data set containing false images.
  Raw data set contains the number of the real errors of image is uncertain, the contrast experiment, in order to study the proportion of the data set contains error image and selection error, the relationship between the image we see some of the tag is set to the wrong image, we set the number of these errors tag is known, so that the image data set error rate can be calculated, Such as the expression (1).
  1 $$ {\text{E}}\_{rate} = \frac{{{\text{ATW}}}}{{{\text{ALL}}}} $$
2. The percentage of true error image number (TW) in the number of possible error image number (MW) screened, which is called Filtering accuracy, is expressed by (Eq. (2)). The higher the value is, the better the algorithm screening effect is. The lower and higher FER is, the better screening effect of the algorithm is.
  2 $$ {\text{F}}\_{acc} = \frac{{{\text{TW}}}}{{{\text{MW}}}} $$
3. The percentage of the number of filtered true error images (TW) in the total number of true error images (ATW) in the data set, known as Filtering recall rate, is denoted by (Eq. (3)). The higher the value is, the stronger the ability of the algorithm to filter true and false images is. In general, the total number of true and false images in a data set is not clear. In order to verify the ability of the algorithm to screen the wrong images, a comparative experiment is designed. We set some wrong image labels in the comparison experiment, and ATW is the total number of wrong images manually set.
  3 $$ {\text{F}}\_{rec} = \frac{{{\text{TW}}}}{{{\text{ATW}}}} $$

### Experimental design and results

Due to esophageal endoscope image error in the data set is mainly within the class label image noise, image is early esophageal carcinoma wrong labeling for esophageal cancer early image, the early carcinoma image error in early image, so the esophagus endoscopic image data set label noise is main kinds of cleaning tags within the noise image. Considering the above characteristics of the esophageal endoscopy image data set, three experiments were designed to verify the validity and feasibility of the proposed method.

Experiment 1: Unknown inaccurate image data set cleaning. This experiment explores whether any given image data set with unknown accuracy and no completely correct training set as the prerequisite can clean label noise and background noise images.

Experiment 2: Cleaning of label noise image in data set class. This experiment explores the influence of the proportion of images containing mislabeled in the dataset on the cleaning accuracy. The specific approach is: in the deleted error image data set, randomly extract the same number of two types of images, respectively, modify their labels to error labels, and then use the model in this paper to automatically screen the error images. For the esophageal endoscopy image data set, 50 images were randomly extracted from the early cancer and non-early cancer data sets and modified with wrong labels (i.e., 100 images of intra-class label noise). In order to verify the generalization ability of the model, we extracted 200, 600, 1000, 2000, 3000 and 4000 in-class tag noise images from the cat and dog image data sets, respectively, and compared the cleaning effect of the model when the data sets contained different proportions of tag noise images.

Experiment 3: Cleaning the noise image of the label outside the data set. The experiment verifies the cleaning accuracy of out-class label noise image and background noise image. To do this, an additional 100 images were added to a dataset of early and non-early esophageal cancer images that had been stripped of true and false images. Among them, 50 images of other esophageal diseases were added to images labeled as early cancer, and 50 images of gastric diseases were added to images labeled as non-early esophageal cancer. Since the newly added 100 images do not belong to the original esophageal endoscopy image set, they belong to the background noise image or the extra-class label noise image of esophageal endoscopy image. In order to verify the generalization ability of the model, we randomly select and add another 2000 human images in the cat and dog image data set, modify their labels as cats or dogs, and then adopt the proposed method to automatically screen these 2000 mislabeled images.

### Cleaning of esophageal endoscopy image data sets

#### Cleaning results of the original image data set of early and non-early esophageal cancer

We cleaned the data set of images of early esophageal cancer and non-early esophageal cancer mentioned in the material. We only cleaned the wrong images in the training set and verification set, and the images in the test set were repeatedly checked by two experienced physicians to ensure that every image was correct. The specific information is shown in Table 3.

**Table 3:** Comparison of esophageal image data sets before and after cleaning.

	Train		
	EEC	N-EEC	Total
Before cleaning	2387	886	3273
After cleaning	2340	876	3216
num	576	215	

**Figure 2:**
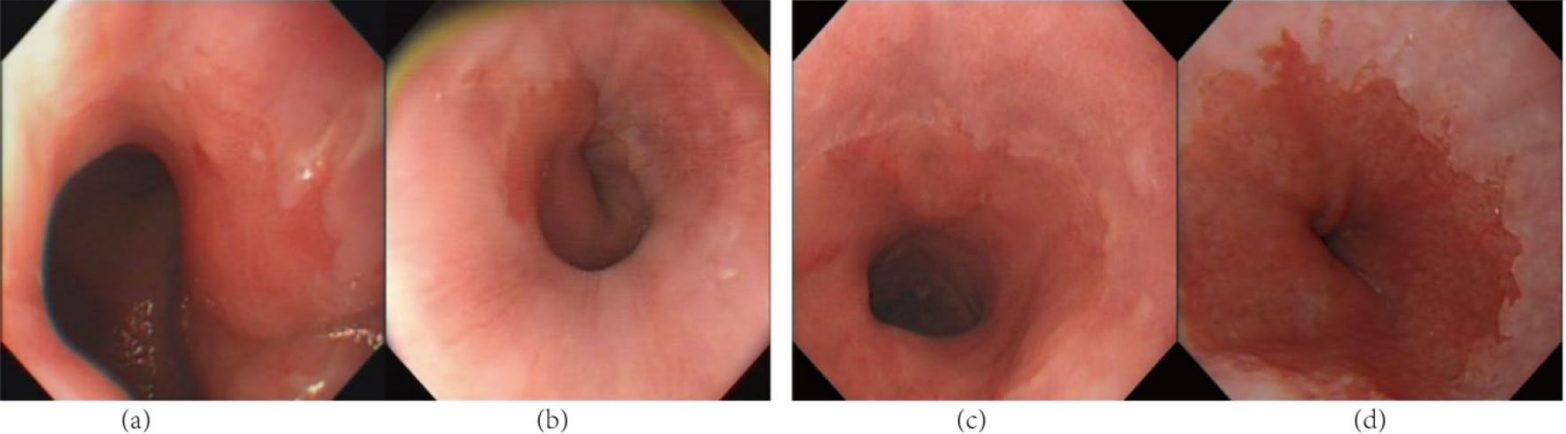
Example of a filtered possible error image. (**a**) True Positive; (**b**) False Negative; (**c**) False Positive; (**d**) True Negative. The screened image is the image with high error rate of each screening and the image cannot be correctly classified by the model.

A total of 57 images with label noise or background noise were screened out, including some mislabeled images and most of the images with unclear or background noise. The 57 images cleaned out included 47 images of early esophageal cancer and 10 images of non-early esophageal cancer. Figure 2 shows an example of a possible error image for filtering.

All possible wrong images automatically screened by the model included some images with correct labels, but those with correct labels might have complex backgrounds or the model could not be correctly classified. Therefore, we deleted all 57 images with possible errors automatically screened, so that there were 3216 images left in the data set. There were 2340 images of early esophageal cancer and 876 images of non-early esophageal cancer.

#### Cleaning results of label noise images within the dataset

After 57 possible wrong images were deleted from the original early cancer data set, there were 3216 images remaining, including 2340 images of early esophageal cancer and 876 images of non-early esophageal cancer. We assumed that the labels of these images were completely correct, and we used these images as the data set of the second experiment.

In order to verify the cleaning effect of intraclass label noise in the esophageal endoscopy image data set, we randomly selected 50 images from both early esophageal cancer and non-early esophageal cancer and modified them with incorrect labels, i.e., 100 intraclass label noise images, and the designed mislabeled images accounted for 3.1% of the total images.

We used the model for automatic screening. After 10 times of automatic screening, a total of 206 images with possible errors were automatically screened out, including 93 images with true errors designed by us, with a screening accuracy of 45% and a recall rate of 93%, accounting for 6.4% of the total images. Therefore, it will not affect the total data set even if all the possible error images screened out automatically are deleted directly without manual screening. It can be concluded from this experiment that our algorithm can effectively screen out the image of in-class tag noise. The specific results are shown in Table 4.

**Table 4:** Esophageal image label error screening results.

	Possible wrong	True wrong	Total design wrong	All	Screening accuracy	Recall
num	192	93	100	3216	48.4%	93%

In the experiment, the mislabeled images designed by us accounted for 3.1% of the total images. Through the automatic screening of the model, we found that the screening accuracy was 48.4%, that is, 48.4% of all possible wrong images screened were wrong images designed by us, and the other images were correct images. The recall rate of wrong images is 93%, and 93% of the wrong images designed by us can be screened out after screening. Therefore, this algorithm can effectively screen out the image with incorrect label within the class.

#### Cleaning results of noise images with labels outside the data set

**Figure 3:**
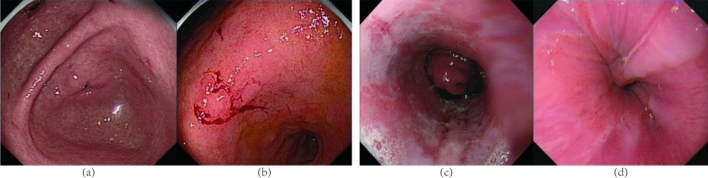
Error images added, wherein (**a**,**b**) are gastric disease images, and (**c**,**d**) are other diseases of endoscopy.

In order to verify the effect of the algorithm on screening noise images of outer label, we randomly extracted 50 images of other esophageal diseases and gastric diseases (as shown in Fig. 3), modified them into labels of images of early esophageal cancer and non-early esophageal cancer respectively, and added them into image data sets of early esophageal cancer and non-early esophageal cancer. The mislabelled dataset contained 3316 images. There were 2390 labels for early cancer and 926 labels for non-early esophageal cancer. Mislabeled images accounted for 3% of the total images.

After 10 times of automatic screening, a total of 302 possible error images were automatically screened out, including 54 of the wrong label images designed by us, with a screening accuracy of 18% and a recall rate of 54%. All possible error images screened accounted for 9% of the total data set images. The specific results are shown in Table 5.

**Table 5:** Filtering results of external label noise images.

	Possible wrong	True wrong	Total design wrong	All	Screening accuracy	Recall
num	302	54	100	3216	18%	54%

Esophagitis and stomach diseases after image change for error label belongs to a class outside label image noise, our algorithm can filter out most of the images of the errors, but compared with the label image noise in class, screening accuracy and recall rate is greatly reduced, so the algorithm in the screening of class labels image noise is relatively difficult, the effect is not ideal.

### Generalization study

To illustrate the generalization capability of the proposed method, experiments 1 to 3 were performed on the Kaggle Computer Vision Contest open dataset.

#### Cleaning results of error images in original cat and dog data sets

The quality of images in cat and dog data sets varies greatly. Some images have low pixels, and some are blocked by other objects. After image data cleaning, original data set is found that about 0.9% of the image is wrong, the part of the error image includes a photo at the same time with dogs and cats, pixel is too low, not a cat dog image, blurred image, etc., after 10 automatic cleaning and manual cleaning, automatic cleaning program makes possible the wrong image about 653, accounting for 2.6% of the total image, Of the 653 images, 221 were found to be true or false after manual cleaning.

When cleaning the wrong data of cat and dog images, it is found that the inclass label noise image and background noise image are easy to be cleaned out, while the out-class label error image is difficult to be cleaned. Some non-cat and non-dog images need to be cleaned for many times, and the effect is not good.

After deleting all true errors and partially blurred images, the remaining images in the dataset are 24,779. Among them, there are 12,370 and 12,409 cat and dog images respectively^30^.

#### Cleaning results of label noise images in cat and dog datasets

The data set used in this experiment is the deleted data set of all the wrong images screened out from the original cat and dog image data set. After deleting the wrong images, there are still 24,779 cat and dog images, and we assume that the labels of these 24,779 cat and dog images are completely correct.

We randomly selected some images with the same number of cats and dogs and changed them to wrong labels, that is, the cat's label was changed to dog and the dog's label was changed to cat. Some in-class label noises were artificially created, and then the algorithm was used for automatic screening. We modified 200, 600, 1000, 2000, 3000, 4000 and 6000 intraclass tag noise images respectively. After the model screening for the same number of times, we found that the less the number of label noise images in the data set, the more difficult the screening. Specific screening results are shown in Table 6.

**Table 6:** Noise image screening results of in-class labels.

ATW	${\text{E}}\_{rate}$ (%)	MW	TW	${\text{F}}\_{acc}$ (%)	${\text{F}}\_{rec}$ (%)
200	0.8	347	186	53.6	93
600	2.4	881	563	64	93.8
1000	4	1335	971	72.7	97.1
2000	8	2278	1908	83.7	95.4
3000	12.1	3269	2836	86.7	94.5
4000	16.1	4385	3844	87.6	96.1
6000	24.2	6684	5682	85	94.7

In this experiment, we designed the inclass tag noise image in the original image to account for 0.8–24.2% of the total image, and then conducted screening. It can be seen from Table 6 that about 95% of in-class tag noise images can be screened out. If the screening batch increases, more tag noise images can be screened out.

**Figure 4:**
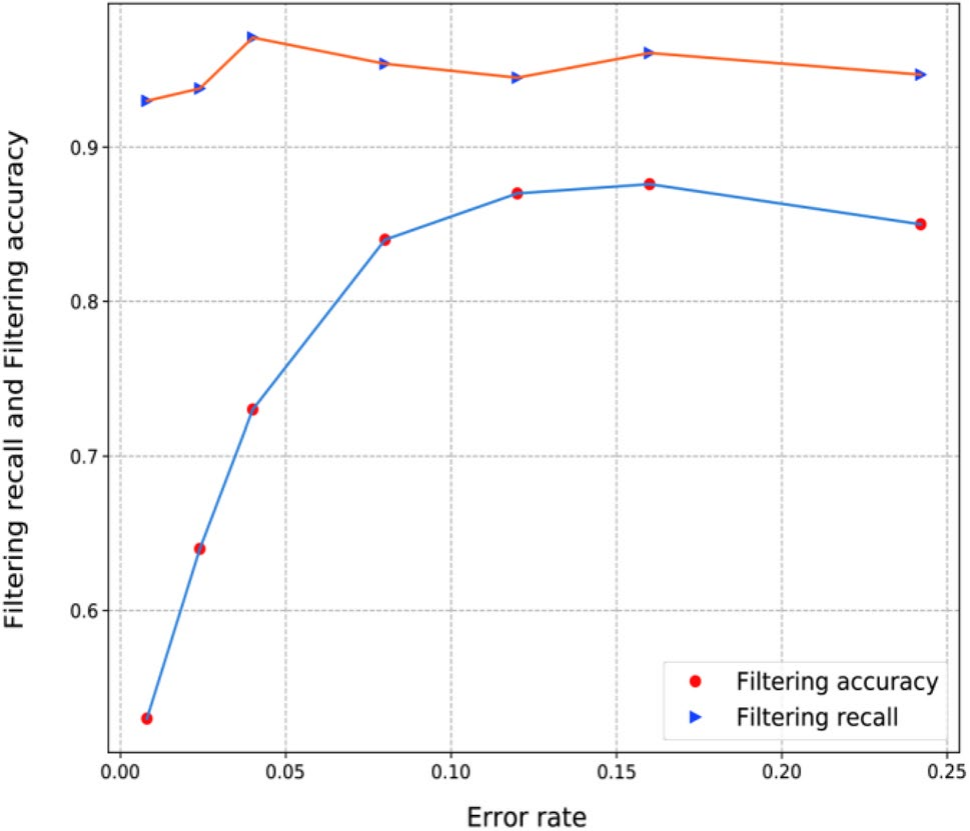
The data set contains the relationship between the ratio of the noise image of the tag within the class and the screening result.

As can be seen from Fig. 4, when the proportion of the data set containing in-class tag noise images is lower than 2%, the screening accuracy is lower than 60%, and the screening is difficult. In all cases, the screening recall rate is above 90%, that is, more than 90% of images with in-class tag noise can be screened out. When the proportion of false images in the image dataset exceeds 20%, the screening accuracy and recall rate decrease.

#### Cleaning results of noise images of out-of-class labels in cat and dog datasets

The data set we used in this experiment was the deleted data set of all the wrong images screened out from the original cat and dog image data set. After deleting the wrong images, there were still 24,779 cat and dog images, and we assumed that the labels of these 24,779 cat and dog images were completely correct. In addition, we searched for 2000 human images and modified them into 1000 cats and 1000 dogs, so that the data set of this experiment contains 26,779 images in total, including 13,409 cats, 13,370 dogs and 2000 extra-class tag noise images.

After screening, we find that if other images are added to the data set, that is, out-of-class tag noise images, this type of tag noise image is difficult to screen. The specific results are shown in Table 7.

**Table 7:** Filtering results of external label noise images.

Total design wrong	Proportion of false images in total	Get possible wrong	True wrong	Screening accuracy	Recall
2000	7.5%	2015	1174	58.3%	58.7%

In this experiment, we randomly selected 2000 human images and modified them into tags of cats and dogs as the noise images of out-class tags for screening. We found that the algorithm was difficult to screen out-class tag noise images with low screening accuracy and recall rate. In addition, after repeated screening experiments, we found that when the proportion of out-of-class label noise images in the total image was less than 2%, the wrong image could not be screened even if the number of screening times was increased.

## Discussion

Because a large number of esophagus endoscopic image data set with high cost and time, plus by subjectivity and experience in the process of manual annotation, fatigue and other factors, it is difficult to obtain large number of accurate data set, so the esophagus endoscopic image data set or is labeled noise or background noise or data set containing the image quantity is small, The characteristic of label noise of esophageal endoscopy image data set is intra class label noise, which is quite different from that of natural image data set. Existing cleaning inaccurate data set methods are mainly concentrated in the natural image data sets, and there is no application to the esophagus endoscopic image inaccurate data set to clean, and most of the data set cleaning method need to completely clean before the training model of data sets to do the premise, and the proposed approach, in front of the training model does not need to completely clean premises a data set, The method presented in this paper can be applied to the cleaning of inaccurate data sets of esophageal endoscopy images and natural images.

The method can effectively clean label image noise in the data set and part of the background noise image, in the process of cleaning of the original data set, because the proportion of the image data set containing the wrong label is unknown, in order to determine the method screening label the effect of noise and background noise of image, we joined the artificial selection steps, After all possible label noise and background noise were automatically screened out by the model, the part of the image was manually screened to determine the effect of the model on label noise and background noise screening. The results showed that images containing tag noise and background noise accounted for about 0.9% of the cat and dog data sets. More than 60% of all possible error images automatically screened by the method in this paper are real label noise and background noise images.

Since most of the label noises of esophageal endoscopy images are mislabeled between the two categories, we call such label noise images intra-class label noise. We set a certain number of in-class label noise images in the clean data set, and then clean them. The second experiment was for the cleaning of intraclass label noise. It can be seen from this experiment that the proposed method has a good effect on the screening of intraclass label noise in the esophageal endoscopy image data set, and about 95% of the intraclass label noise can be screened out. In the generalization experiment (Table 6), we find that the proposed method also has a good screening effect on the natural image data set of in-class tag noise, and has a good screening effect on the image data set of in-class tag noise with an error rate of less than 24%.

We added some other images to the cleaned data set, and modified the other images into one of the two types of labels to create some noise images of out-class labels. The method in this paper can only screen 50–60% of the out-of-class tag noise and background noise images. Further research is needed on the screening of out-of-class label noise.

Our method has achieved good results in cleaning label noise images and background noise images in the early esophageal cancer data set and natural image data set, and our method does not require a completely clean data set before cleaning the data set.

However, the method in this paper still has some limitations. First, our method removes a small number of correctly labeled images while deleting label noise and background noise images. Secondly, our method has not been extended to the cleaning of label noise and background noise images in multi-classification datasets. Finally, the cleaning effect of our method is not good in cleaning the noise images and background noise images of out-of-class labels, and further study is needed.

## Conclusion

In this study, we propose a method for cleaning label noise and background noise images from endoscopy images in inaccurate supervised learning, which can be trained on data containing label noise and background noise. We design a new neural network VGG_NIN and error image screening module, which can automatically screen out all possible error images in the data set. Among the screened possible error images, the true error images account for 60–87%. The experimental results show that this method can effectively clean the label noise and background noise images in the esophageal endoscopy image data set and natural image data set, which is the first application in cleaning the label noise and background noise images in the esophageal endoscopy image data set.

In future work, we intend to perform cleaning label noise and background noise images in the multi-classification data set of esophageal endoscopy images and natural images, and improve the neural network model to improve the cleaning speed.
